# Supplementary material for: Range reduction of Oblong Rocksnail, Leptoxis compacta, shapes riverscape genetic patterns
Source: PeerJ. 2020 Sep 1;8:e9789. doi: 10.7717/peerj.9789 (PMC7473045; doi:10.7717/peerj.9789)
Supplement: Supplemental Information 1 [file peerj-08-9789-s001.docx]

Supplementary Table 1: Collection localities, Auburn University Museum of Natural History, and SRA accession numbers.

| Lab Number | Locality | AUMNH Catalog Number | NCBI SRA accession |
| --- | --- | --- | --- |
| Lcom_pop01 | Cahaba River at old Marvel slab | 45652 | SRR11773571 |
| Lcom_pop02 | Cahaba River at old Marvel slab | 45653 | SRR11773570 |
| Lcom_pop03 | Cahaba River at old Marvel slab | 45654 | SRR11773559 |
| Lcom_pop04 | Cahaba River at old Marvel slab | 45655 | SRR11773548 |
| Lcom_pop05 | Cahaba River at old Marvel slab | 45656 | SRR11773537 |
| Lcom_pop06 | Cahaba River at old Marvel slab | 45657 | SRR11773604 |
| Lcom_pop08 | Cahaba River at old Marvel slab | 45658 | SRR11773593 |
| Lcom_pop09 | Cahaba River at old Marvel slab | 45659 | SRR11773582 |
| Lcom_pop10 | Cahaba River at old Marvel slab | 45660 | SRR11773573 |
| Lcom_pop11 | Cahaba River at old Marvel slab | 45661 | SRR11773572 |
| Lcom_pop12 | Cahaba River at old Marvel slab | 45662 | SRR11773569 |
| Lcom_pop13 | Cahaba River at old Marvel slab | 45663 | SRR11773568 |
| Lcom_pop14 | Cahaba River at old Marvel slab | 45664 | SRR11773567 |
| Lcom_pop15 | Cahaba River at old Marvel slab | 45665 | SRR11773566 |
| Lcom_pop16 | Cahaba River at old Marvel slab | 45666 | SRR11773565 |
| Lcom_pop17 | Cahaba River at old Marvel slab | 45667 | SRR11773564 |
| Lcom_pop18 | Cahaba River at old Marvel slab | 45668 | SRR11773563 |
| Lcom_pop19 | Cahaba River at old Marvel slab | 45669 | SRR11773562 |
| Lcom_pop20 | Cahaba River at old Marvel slab | 45670 | SRR11773561 |
| Lcom_pop21 | Cahaba River at above Shades Creek | 45671 | SRR11773560 |
| Lcom_pop22 | Cahaba River at above Shades Creek | 45672 | SRR11773558 |
| Lcom_pop23 | Cahaba River at above Shades Creek | 45673 | SRR11773557 |
| Lcom_pop24 | Cahaba River at above Shades Creek | 45674 | SRR11773556 |
| Lcom_pop25 | Cahaba River at above Shades Creek | 45675 | SRR11773555 |
| Lcom_pop26 | Cahaba River at above Shades Creek | 45676 | SRR11773554 |
| Lcom_pop27 | Cahaba River at above Shades Creek | 45677 | SRR11773553 |
| Lcom_pop28 | Cahaba River at above Shades Creek | 45678 | SRR11773552 |
| Lcom_pop29 | Cahaba River at above Shades Creek | 45679 | SRR11773551 |
| Lcom_pop30 | Cahaba River at above Shades Creek | 45680 | SRR11773550 |
| Lcom_pop31 | Cahaba River at above Shades Creek | 45681 | SRR11773549 |
| Lcom_pop32 | Cahaba River at above Shades Creek | 45682 | SRR11773547 |
| Lcom_pop33 | Cahaba River at above Shades Creek | 45683 | SRR11773546 |
| Lcom_pop34 | Cahaba River at above Shades Creek | 45684 | SRR11773545 |
| Lcom_pop35 | Cahaba River at above Shades Creek | 45685 | SRR11773544 |
| Lcom_pop36 | Cahaba River at above Shades Creek | 45686 | SRR11773543 |
| Lcom_pop37 | Cahaba River at above Shades Creek | 45687 | SRR11773542 |
| Lcom_pop38 | Cahaba River at above Shades Creek | 45688 | SRR11773541 |
| Lcom_pop39 | Cahaba River at above Shades Creek | 45689 | SRR11773540 |
| Lcom_pop40 | Cahaba River at above Shades Creek | 45690 | SRR11773539 |
| Lcom_pop41 | Cahaba River at Booth's Ford | 45691 | SRR11773538 |
| Lcom_pop42 | Cahaba River at Booth's Ford | 45692 | SRR11773536 |
| Lcom_pop44 | Cahaba River at Booth's Ford | 45693 | SRR11773613 |
| Lcom_pop45 | Cahaba River at Booth's Ford | 45694 | SRR11773612 |
| Lcom_pop46 | Cahaba River at Booth's Ford | 45695 | SRR11773611 |
| Lcom_pop47 | Cahaba River at Booth's Ford | 45696 | SRR11773610 |
| Lcom_pop48 | Cahaba River at Booth's Ford | 45697 | SRR11773609 |
| Lcom_pop49 | Cahaba River at Booth's Ford | 45698 | SRR11773608 |
| Lcom_pop50 | Cahaba River at Booth's Ford | 45699 | SRR11773607 |
| Lcom_pop51 | Cahaba River at Booth's Ford | 45700 | SRR11773606 |
| Lcom_pop52 | Cahaba River at Booth's Ford | 45701 | SRR11773605 |
| Lcom_pop53 | Cahaba River at Booth's Ford | 45702 | SRR11773603 |
| Lcom_pop54 | Cahaba River at Booth's Ford | 45703 | SRR11773602 |
| Lcom_pop55 | Cahaba River at Booth's Ford | 45704 | SRR11773601 |
| Lcom_pop56 | Cahaba River at Booth's Ford | 45705 | SRR11773600 |
| Lcom_pop57 | Cahaba River at Booth's Ford | 45706 | SRR11773599 |
| Lcom_pop58 | Cahaba River at Booth's Ford | 45707 | SRR11773598 |
| Lcom_pop59 | Cahaba River at Booth's Ford | 45708 | SRR11773597 |
| Lcom_pop60 | Cahaba River at Booth's Ford | 45709 | SRR11773596 |
| Lcom_pop61 | Cahaba River at Lebron canoe launch | 45710 | SRR11773595 |
| Lcom_pop62 | Cahaba River at Lebron canoe launch | 45711 | SRR11773594 |
| Lcom_pop63 | Cahaba River at Lebron canoe launch | 45712 | SRR11773592 |
| Lcom_pop64 | Cahaba River at Lebron canoe launch | 45713 | SRR11773591 |
| Lcom_pop65 | Cahaba River at Lebron canoe launch | 45714 | SRR11773590 |
| Lcom_pop66 | Cahaba River at Lebron canoe launch | 45715 | SRR11773589 |
| Lcom_pop67 | Cahaba River at Lebron canoe launch | 45716 | SRR11773588 |
| Lcom_pop68 | Cahaba River at Lebron canoe launch | 45717 | SRR11773587 |
| Lcom_pop69 | Cahaba River at Lebron canoe launch | 45718 | SRR11773586 |
| Lcom_pop70 | Cahaba River at Lebron canoe launch | 45719 | SRR11773585 |
| Lcom_pop71 | Cahaba River at Lebron canoe launch | 45720 | SRR11773584 |
| Lcom_pop72 | Cahaba River at Lebron canoe launch | 45721 | SRR11773583 |
| Lcom_pop73 | Cahaba River at Lebron canoe launch | 45722 | SRR11773581 |
| Lcom_pop74 | Cahaba River at Lebron canoe launch | 45723 | SRR11773580 |
| Lcom_pop75 | Cahaba River at Lebron canoe launch | 45724 | SRR11773579 |
| Lcom_pop76 | Cahaba River at Lebron canoe launch | 45725 | SRR11773578 |
| Lcom_pop77 | Cahaba River at Lebron canoe launch | 45726 | SRR11773577 |
| Lcom_pop78 | Cahaba River at Lebron canoe launch | 45727 | SRR11773576 |
| Lcom_pop79 | Cahaba River at Lebron canoe launch | 45728 | SRR11773575 |
| Lcom_pop80 | Cahaba River at Lebron canoe launch | 45729 | SRR11773574 |
