# Supplementary material for: Range reduction of Oblong Rocksnail, Leptoxis compacta, shapes riverscape genetic patterns
Source: PeerJ. 2020 Sep 1;8:e9789. doi: 10.7717/peerj.9789 (PMC7473045; doi:10.7717/peerj.9789)

Supplementary Figure 1: Pairwise co-ancestry matrix and simple tree inferred with fineRADstructure. Boxes surround the two main groupings. Tick marks represent individuals. Lcompop_01-20: Cahaba River at old Marvel slab; Lcompop_21-40: Cahaba River above Shades Creek; Lcompop_41-60: Cahaba River at Booth's Ford; Lcompop_61-80: Cahaba River at canoe launch.

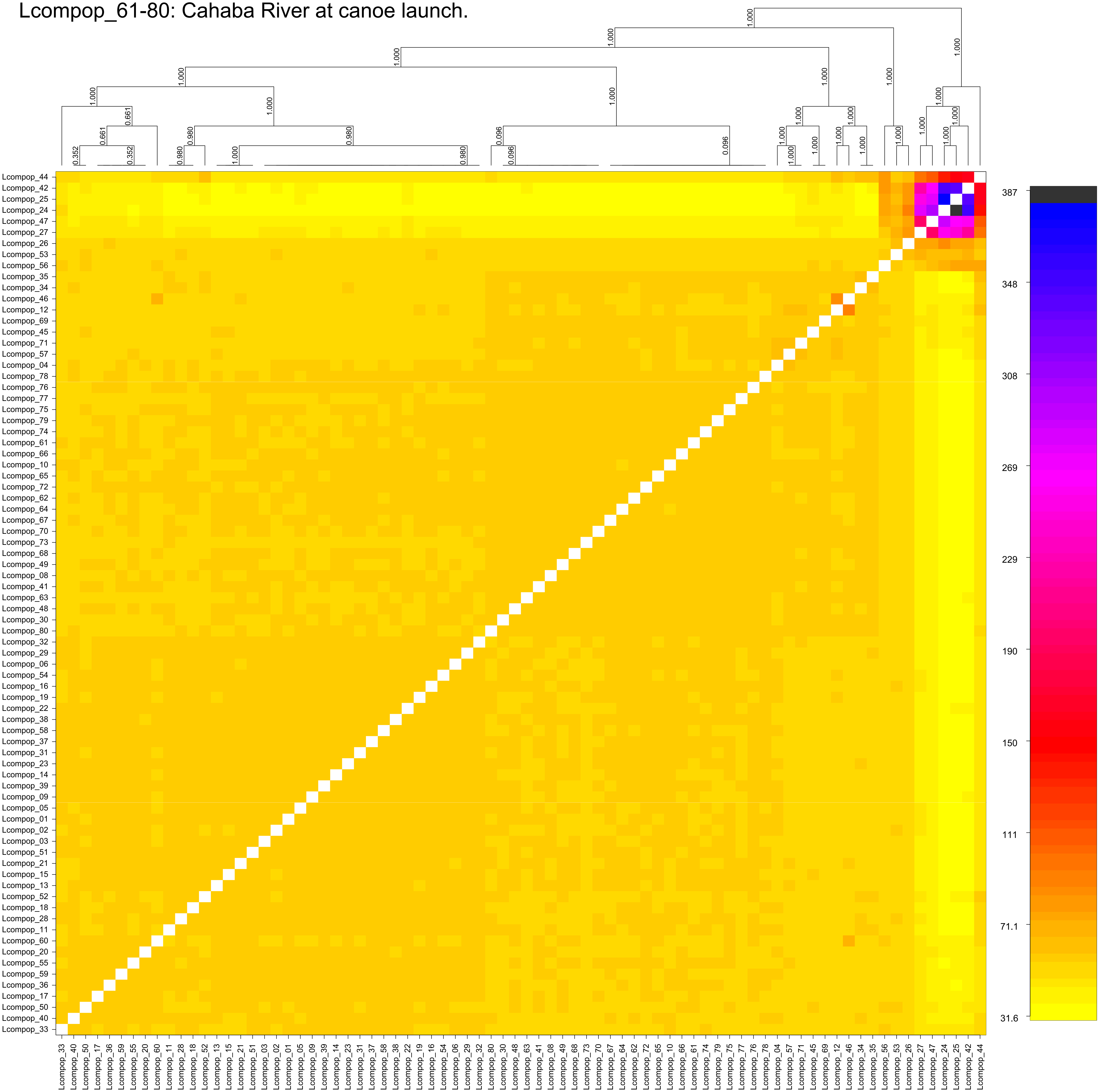

Supplement: Supplemental Information 2 — Tick marks represent individuals. Lcompop_01-20: Cahaba River at old Marvel slab; Lcompop_21-40: Cahaba River above Shades Creek; Lcompop_41-60: Cahaba River at Booth’s Ford; Lcompop_61-80: Cahaba River at canoe launch. [file peerj-08-9789-s002.pdf]
